# A Query

**Published:** 1887-12

**Authors:** 


### A QUERY.

*Editors of the Record:* Is Podophyllin a chologogue, and can it be relied upon as a substitute for calomel? A SUBSCRIBER.

To the above question we reply that certain experiments upon dogs by Professor Rutherford, would seem to show that podophyllin excites the liver to secretion, while the Eclectics and many regular practitioners are in the habit of using the remedy as a cholagogue, claiming that it acts upon the liver, and may take the place of calomel in many instances.

Our own view of this drug—confirmed by our own experience during many years of practice—is, that *it is not a cholagogue*, and that it is by no means a substitute for calomel. Its apparent action upon the liver is due to its action upon the duodenum, the peristalsis of which it stimulates and assists the discharge of bile by releiving the torpor of the upper bowel, removing congestion of the mucous membrane, and thus enabling the orifices of the bile and pancreatic ducts to discharge freely their contents. This, of course, is a valuable property; but the drug does not act specially upon the liver so as to stimulate the biliary secretion. That it may often be used with benefit in that class of biliary disorders which are due to torpor of the duodenum and to obstruction of the bile and pancreatic ducts resulting from such torpor, we admit to be true. The same may be said of many other purgatives which act upon the duodenum. Senna is one of this class of drugs, and is even better than podophyllin. It is to the senna in Simmons' famous liver medicine that is due all its reputation as a cholagogue. Calomel, however, has a specific property upon the liver, due not so much to its action upon the peristalsis, which in some degree it also possesses, but to a property which it exerts upon the gland itself. It not only exerts this specific power upon this largest of glands, but has a special influence also upon all other glands in the system. When pushed to a certain extent, this specific action is directed with greatest force upon the salivary glands, causing profuse secretion and producing what we call salivation. The pancreas being of like structure to the salivary glands is doubtless also acted upon by calomel. So the glands of Brunner in the duodenum, which in function are supplementary to the salivary glands, are doubtless also stimulated by calomel.

If we are right in these views—and certainly we are sustained, both by theory and by facts—we can find in them a clue to the explanation of the wonderful powers of calomel as a medicinal agent. W.

*Queries.*—Above we have answered a query in regard to podophyllin, which may prove suggestive and important to medical brethren upon the same subject; and we would be glad to see a larger spirit of inquiry and progress on the part of our readers. Let our friends ask questions on medical subjects, and some of our subscribers, if not the editors, may be able to answer, and thus by interchanging views, we may be mutually instructed and interested. We wish, therefore, our brethren to ask and also to answer questions. We shall in this and other ways endeavor to increase the usefulness of our journal next year, and kindly ask the aid and co-operation of our readers, not only in the matter of writing, but also in extending the circulation of the RECORD.

